# Fall Risk Assessment and Early-Warning for Toddler Behaviors at Home

**DOI:** 10.3390/s131216985

**Published:** 2013-12-10

**Authors:** Mau-Tsuen Yang, Min-Wen Chuang

**Affiliations:** Department of Computer Science & Information Engineering, National Dong-Hwa University, No. 1, Sec. 2, Da-Hsueh Rd., Shoufeng, Hualien 974, Taiwan; E-Mail: 610121011@ems.ndhu.edu.tw

**Keywords:** Kinect, toddler, childcare, fall risk, early-warning

## Abstract

Accidental falls are the major cause of serious injuries in toddlers, with most of these falls happening at home. Instead of providing immediate fall detection based on short-term observations, this paper proposes an early-warning childcare system to monitor fall-prone behaviors of toddlers at home. Using 3D human skeleton tracking and floor plane detection based on depth images captured by a Kinect system, eight fall-prone behavioral modules of toddlers are developed and organized according to four essential criteria: posture, motion, balance, and altitude. The final fall risk assessment is generated by a multi-modal fusion using either a weighted mean thresholding or a support vector machine (SVM) classification. Optimizations are performed to determine local parameter in each module and global parameters of the multi-modal fusion. Experimental results show that the proposed system can assess fall risks and trigger alarms with an accuracy rate of 92% at a speed of 20 frames per second.

## 1. Introduction

Toddlers are likely to fall because their heads are heavier in proportion to the rest of their bodies and they are still learning how to find their balance at this stage. According to Morrongiello's study [1], falls are the most common cause of serious injury in toddlers. Moreover, toddlers and preschoolers experience most fall injuries at home. Consequently, fall detection, prediction, and prevention to assist parents' supervision become critical issues for toddler healthcare at home.

Starting from a balanced state, a typical human fall usually involves the transition of a series of states: losing balance, impacting with objects or the floor, and finally lying down after the impact. In contrast to fall detection (detect after lying down) [2–7] and pre-impact detection (detect descending in just a few milliseconds before the first impact) [8–11], we propose an early-warning system to identify fall-prone behaviors, assess fall risks, and predict potential fall dangers in the relatively long-term future (a few seconds). It is extremely important to trigger an alarm early so toddlers are alerted to stop behaving dangerously and caregivers have time to intervene to avoid accidental falls. However, providing early-warning of fall risks poses significant computational difficulties and multi-modal fusion problems, particularly in the case of toddlers, who are relatively vivacious and energetic compared to adults. Moreover, making an accurate and quick decision is critical in a fall risk assessment system. Missed and late detections can lead to dangerous situations, and false alarms can cause users to lose trust in a system and ignore system alerts.

**Figure 1.**
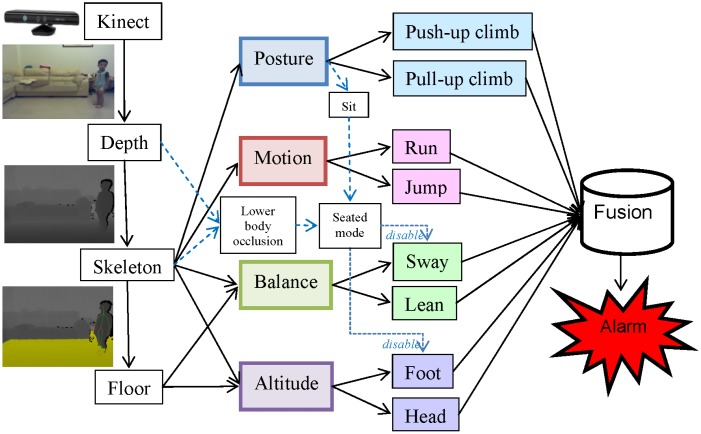
Flowchart of the proposed fall risk assessment and early-warning system.

Fall risks can be influenced by intrinsic, environmental, and behavioral factors. The intrinsic factors include individual health conditions and medical records. The environmental factors involve potential dangers found in a living space, such as a slippery floor, floor clutter, or furniture with sharp edges. The behavioral factors involve body postures or movements that may lead to falls. This paper focuses on the identification of these behavioral factors by monitoring the actions of toddlers using a Kinect [12] at home. Distinguishing fall-prone behaviors from other activities of daily life (ADL) accurately is the key issue in establishing an effective fall risk model. Particularly, four essential criteria (posture, motion, balance, and altitude) were applied to design eight fall-prone behavioral modules of toddlers. The final assessment was generated by a multi-modal fusion using either a weighted mean thresholding or a support vector machine (SVM) [13] classification. A local optimization was applied to determine the parameter inside each module, and a global optimization was performed to determine the parameters for the multi-modal fusion. Figure 1 shows the hierarchical framework of the proposed fall risk assessment system for toddler behaviors at home.

The remaining parts of this paper are organized as follows: Section 2 provides the literature review. Section 3 explains the fall risk assessments using various independent modules. Section 4 presents the local and global parameter optimizations and proposes two schemes for the multi-modal fusion of fall risks. Section 5 discusses the experimental setup and comparison results, and Section 6 offers a conclusion.

## 2. Background

Various wearable sensors, such as accelerometers and gyroscopes, have been proposed to detect elderly falls [2,3] for making automatic emergency calls. To reduce the severity of injury, a few approaches tried to detect a fall in its descending phase before the first impact [8,10,11]. With the capability of the pre-impact fall detection, a wearable airbag or inflatable device can be triggered to provide impact protection [9]. Table 1 compares various approaches using wearable inertial sensors for fall and pre-impact detections. Approaches using a tri-axis accelerometer [2,8] can measure magnitudes and directions of 3D vibrations. The bias caused by the gravity can be cancelled by a calibration process. Subsequently, a rough 3D translation can be approximated through double integrals of the measured accelerations. Approaches using a gyroscope [3] were capable of measuring angular velocity that can be further aggregated to obtain an angular displacement through a single integral. Combining the individual characteristics of the accelerometers and gyroscopes in an inertial measurement unit (IMU), the latest hybrid approaches [9–11] can detect falls or pre-impacts more reliably. However, the requirement of wearing the equipment was intrusive and increased the fall risk itself.

In contrast, cameras installed at home can provide a non-intrusive way for fall detection. Table 2 compares fall detectors using different types of cameras including infrared cameras, color cameras, depth cameras, and Kinects. For methods using infrared cameras, Sixsmith and Johnson [4] developed an intelligent fall detector, called SIMBAD, based on neural networks and a low-cost array of infrared detectors. Tao *et al.* [5] utilized an infrared ceiling sensor network and SVM to recognize eight activities including walking, tidying, watching, reading, taking, using PC, lying, and sweeping. For methods using a typical color camera, Rougier *et al.* [6] detected simulated falls of seniors based on motion history images (MHI) and human shape variations. Na *et al.* [14] presented a vision-based toddler tracking system that performed regional merges and splits to handle partial visual occlusions. Fall risk factors were identified by detecting floor clutter and checking if a toddler moved near or leaving the floor area boundary. Apart from fall detections, Nomori *et al.* [15] trained an infant climbing control model by putting a set of rectangular parallelepipeds with various sizes in the daily living space.

For methods using a depth camera, Lee and Chung [16] exploited depth information for fall detections based on the analysis of shape features and 3D trajectories. Because the depth information was invariant to the existence of shadow, the problem of shadow removal was also addressed. Diraco *et al.* [7] proposed an elderly fall detection system based on an active depth camera. After a self-calibration process, a floor plane was detected and a human skeleton was extracted to recognize four postures: lying, sitting, standing, and bending. For methods using a Kinect, Ni *et al.* [17] presented a get-up event detector to prevent potential falls in hospitals based on RGBD images captured by a Kinect. Features of MHI, histogram of oriented gradients (HOG), and histogram of optic flows (HOF) were extracted and combined through a multiple kernel learning. Except for fall detections, Mozos *et al.* [18] utilized a mobile robot equipped with a Kinect to categorize indoor places including corridor, kitchen, laboratory, study room, and office. Unlike these methods, we proposed an early-warning childcare system to assess fall risks by monitoring eight fall-prone behaviors of toddlers using a Kinect at home. A multi-modal fusion was carried out to integrate fall risk measurements from eight behavioral modules in four distinct criteria for the alarm triggering.

## 3. Fall Risk Assessments for Four Distinct Modules

To model toddler behaviors in daily life at home, 160 video clips (each clip being 3 s in duration) containing normal ADL or fall-prone actions were captured. To acquire the ground truths of fall risks, each video was evaluated by a childcare expert using the questionnaire in Table 3. Based on manual categorization, Table 4 showed five typical safe and another five typical fall-risky types of toddler's behaviors. For each type of behavior, the childcare expert identified the criteria that were useful to classify the behavior type as safe or fall-risky, for example, upright posture and good balance were useful criteria to classify “jumping rope” as safe; high motion and high altitude were useful criteria to classify “high-jumping” as fall-risky. The analysis of these toddler behaviors was helpful in identifying the essential criteria (posture, motion, balance, and altitude) for fall risk assessments.

**Figure 2.**
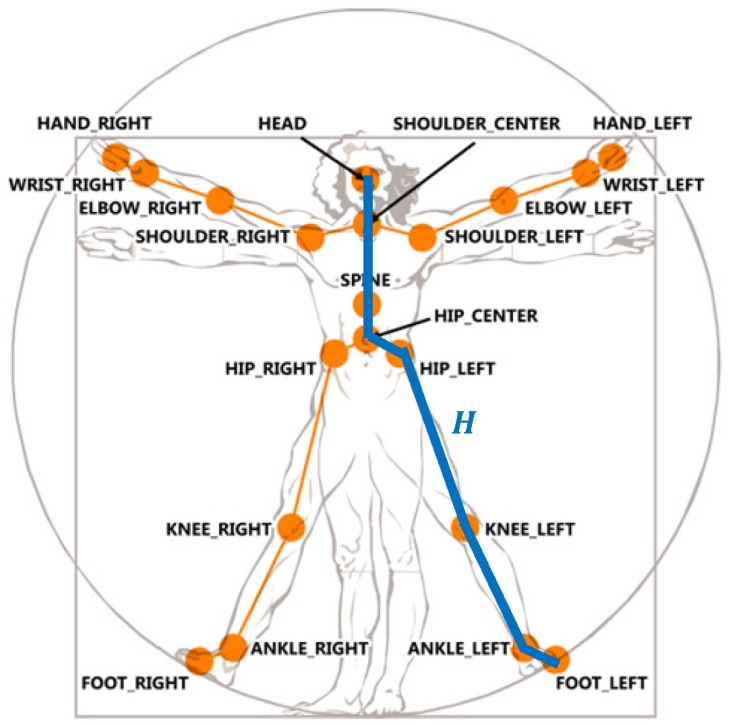
Names and locations of the twenty skeletal joints in a Kinect [12]. Blue line segments represent the set of bones used for body height estimation.

Depth imaging technology has advanced dramatically over the last few years. The latest consumer market depth camera, Kinect, was proven practical in various applications. The proposed toddler fall risk assessment system employed the Kinect which captured both color and depth images at thirty frames per second with 640 × 480 resolution. A pixel in a depth image indicated the calibrated depth of a 3D point in the scene. Each depth image was segmented into a dense probabilistic body part labeling by dividing a human body into thirty-one parts [19]. The body parts were defined to be spatially localized near twenty skeletal joints, hence the 3D locations of the skeletal joints can be determined by back-projecting these inferred parts into a world space. As shown in Figure 2, a complete skeleton was recorded in a sixty-dimensional vector containing 3D coordinates of twenty skeletal joints. A stream of the skeletal vectors over time was taken as the input data for fall risk assessment in multiple criteria: posture, motion, balance, and altitude.

### 3.1. Posture

**Figure 3.**
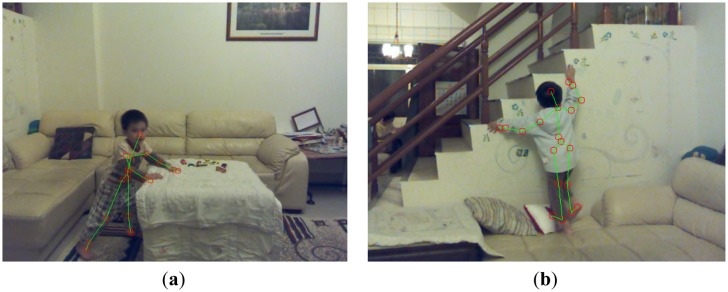
Posture module (**a**) push-up climb and (**b**) pull-up climb.

Static human postures provide important clues for fall risks and can be recognized without using temporal information. In particular, we focused on recognizing the most fall-prone posture: the climbing. SVM is widely used to recognize static patterns by using a supervised learning. A nonlinear SVM can transform the input space to a higher dimensional space and construct a hyperplane to separate two classes in the transformed space. As shown in Figure 3, climbing postures can be divided to two kinds: the push-up climb (pushing a lower object by hands to raise the human body) and the pull-up climb (pulling a higher object by hands to raise the human body). Two kinds of climbing postures significantly differed in nature, so they were separately modeled by distinct SVMs. In our experiments, ninety frames with push-up climb postures, ninety frames with pull-up climb postures, and ninety frames with other postures were used to train two SVMs of the push-up climb and the pull-up climb individually.

To handle the scaling problem, a standard normalization was performed on the stream of skeletal vectors by subtracting the mean and then dividing by the standard deviation in each feature dimension. For the *j*-th frame in the training set with *l* frames (*l* = 90 in this case), a sixty-dimensional vector *x_j_* contained the 3D coordinates of twenty skeletal joints and a Boolean flag *y_j_* indicated the ground truth of whether the frame contained a climbing posture:

(1) $$y_{j}=\{\begin{array}{ll} +1 & ,\text{if}\;j-\text{th frame contains a climbing posture}\\ -1 & ,\text{otherwise} \end{array}$$

Given a set of labeled pairs (*x_j_*, *y_j_*), the SVM became an optimization problem to find a separating hyperplane with the maximum margin. The margins of the hyperplane can be described by the following equations:

(2) $$\{\begin{array}{c} \begin{array}{ccc} w\cdot x_{j}-b\geq +1 & \text{if} & y_{j}=+1 \end{array}\\ \begin{array}{ccc} w\cdot x_{j}-b\leq -1 & \text{if} & y_{j}=-1 \end{array} \end{array}$$

The search of the parameters (*w*, *b*) of the optimal hyperplane can be represented by a quadratic programming optimization:

(3) $$\begin{array}{ccc} \underset{\left(w,b\right)}{\min}\frac{1}{2}{\left\|w\right\|}^{2} & \text{subject to} & y_{j}(w\cdot x_{j}-b)\geq 1 \end{array}$$

The Karush-Kuhn-Tucker condition implied that the solution can be expressed as a linear combination of the training vectors, *i.e.*, $w=\overset{n}{\underset{j=1}{\text{∑}}}\alpha_{j}y_{j}x_{j}$ where *α_j_* were positive real numbers that maximize the following terms:

(4) $$\begin{array}{ccc} \underset{\left(\alpha_{j}\right)}{\max}\left[\overset{n}{\underset{j=1}{\text{∑}}}\alpha_{j}-\frac{1}{2}\underset{\left(j,k\right)}{\text{∑}}\alpha_{j}\alpha_{k}y_{j}y_{k}K(x_{j},x_{k})\right] & \text{subject to} & \alpha_{j}\geq 0\;\text{and}\;\overset{n}{\underset{j=1}{\text{∑}}}\alpha_{j}\;y_{j}=0 \end{array}$$

The training vectors *x_j_* were transformed into a higher dimensional space by a kernel function *K*. The effectiveness of SVM depended on the selection of the kernel. After experimenting a linear kernel and various nonlinear kernels, we found that the quadratic polynomial provided the most accurate classification of the climbing posture:

(5) $$K(x_{j},x_{k})={\left(x_{j}x_{k}+1\right)}^{2}$$

The final decision function was $f(x)=\text{sign}(\overset{n}{\underset{j=1}{\text{∑}}}\alpha_{j}y_{j}K(x_{j},x)-b)$, *i.e.*, if the decision function *f*(*x*) returned a positive value, the frame with skeletal feature *x* was classified as a climbing posture; otherwise, it was classified as a non-climbing posture.

Furthermore, a performance-based method was applied for feature reduction in the training stage of SVM. If removing a feature improved the overall accuracy rate of cross-validation, the feature was considered redundant; if removing a feature degraded the overall accuracy rate of cross-validation, the feature was considered important. An iterative procedure was performed to improve the accuracy rate by removing one redundant feature at a time until no feature could be removed without a loss of accuracy. We found that the accuracy rate can be improved (from 88% to 90%) with a reduced set of features (from 60 to 55) in the SVM classification of the climbing posture.

In addition to the posture classifications, a confidence measure of each classification was required for fall risk assessment. According to Platt's work [20], a signed distance *d* between a feature point and the SVM's hyperplane can be used as the confidence measure of the classification. The bigger the positive distance *d* was, the more probable the person was in a climbing state, and the higher the fall risk should be. Thus, the fall risk related to the push-up climbing state *p* (*push*_*up*_*climb*) was calculated as:

(6) $$p(\text{push}\_up\_\text{climb})=\{\begin{array}{ll} 1-e^{-d^{2}/\alpha_{1}} & ,\text{if}\;d>0\\ 0 & ,\text{otherwise} \end{array}$$

Similarly, the fall risk based on the pull-up climbing state *p*(*pull*_*up*_*climb*) was evaluated as:

(7) $$p(\text{pull}\_up\_\text{climb})=\{\begin{array}{ll} 1-e^{-d^{2}/\alpha_{2}} & ,\text{if}\;d>0\\ 0 & ,\text{otherwise} \end{array}$$

Besides, a posture of sitting was trained and classified using a SVM in the same manner. As shown in Figure 1, the classification result of the sitting posture did not directly contribute to the fall risk but was employed to trigger a seated mode that disabled the body sway and foot altitude modules as described later in Sections 3.3 and 3.4.

### 3.2. Motion

Human motion analysis plays an essential role in fall predictions. Though it is impossible to completely stop toddlers from running and jumping, toddlers can easily trip on objects left on the floor when they run too fast of jump too high. We proposed to detect dangerous motions such as rush-running and high-jumping by considering temporal information. Hidden Markov Model (HMM) [21] is well-known for applications in dynamic pattern recognition. However, a typical HMM contains a large number of parameters that need to be trained using a big amount of sample videos. As an alternative, statistics of kinematic parameters of human body motion were analyzed. After comparing several simple dynamic features such as mean and variance of velocity and acceleration, we found that the mean of the floor plane projected body velocity and variance of the vertical coordinate of the body centroid were the dominating features that can well distinct rush-running and high-jumping from other ADL.

**Figure 4.**
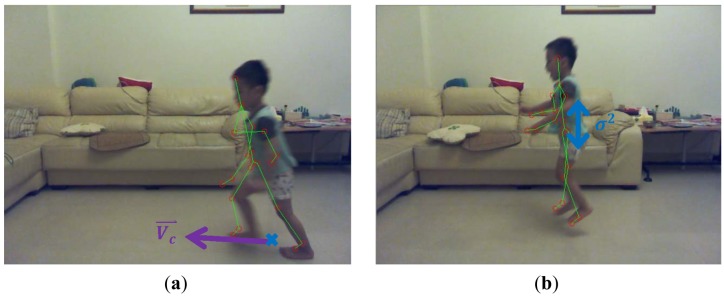
Motion module (**a**) rush-running and (**b**) high jumping.

A skeletal centroid was defined as the 3D mean location of the distribution of the body mass in 3D space, and can be approximated by computing the center of mass of the nineteen bones in the skeleton. A 3D floor plane was detected by the Random Sample Consensus (RANSAC) [22] algorithm based on the depth image. Supposing that the 3D skeletal centroid projected onto the 3D point *P_c_* on the floor plane as shown in Figure 4a, a 3D ground-projected velocity *V_c_*, or called motion vector, can be obtained by a temporal subtraction of two ground-projected centroids in 3D between consecutive frames. The bigger the magnitude of the ground-projected velocity ‖*V_c_*‖ was, the more probable the person was in a running state, and the higher the fall risk should be. Accordingly, the fall risk related to the rush-running state *p*(*run*) was measured as:

(8) $$p(\text{run})=1-e^{-{\left\|V_{c}\right\|}^{2}/\alpha_{3}}$$

Similarly, a temporal variance σ*^2^* of the vertical coordinate of the 3D skeletal centroid can be measured across a sliding window of frames. As shown in Figure 4b, the bigger the temporal variance σ*^2^* of the 3D centroid's *y*-coordinate was, the more probable the person was in a jumping state, and the higher the fall risk should be. Thus, the fall risk based on the high-jumping state *p*(*jump*) was estimated as:

(9) $$p(\text{jump})=1-e^{-\sigma^{2}/\alpha_{4}}$$

### 3.3. Balance

**Figure 5.**
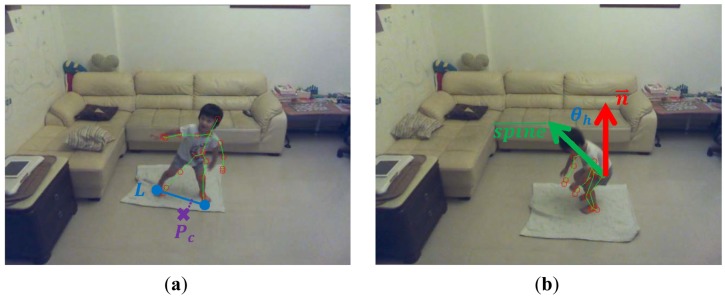
Balance module (**a**) body sway and (**b**) body lean.

Toddlers are still learning about balance, so imbalance features provide important signs of fall dangers. Fall risks can be assessed as the level of sway and lean of the human body. The body sway referred to the body movements made by an individual to maintain a balanced position, and was measured by the displacement between the ground-projected body centroid and the base of support on the floor plane. As shown in Figure 5a, the longer the distance between the body centroid projection and the base of support was, the higher the fall risk should be. Supposing that the skeletal joint FOOT_LEFT projected onto the floor plane point with coordinate (*x_l_*, *z_l_*) and the skeletal joint FOOT_RIGHT projected onto the floor plane point with coordinate (*x_r_*, *z_r_*), a 2D base-of-support line *L* on the floor plane representing the base of support was defined using the following equation:

(10) $$\left(x_{r}-x_{l}\right)z-\left(z_{r}-z_{l}\right)x+\left(z_{r}-z_{l}\right)x_{l}-\left(x_{r}-x_{l}\right)z_{l}=0$$

Supposing that the skeletal centroid projected onto the floor plane point *P_c_* with coordinate (*x_c_*, *z_c_*), the distance from the point *P* to the base-of-support line *L* was computed using the following equation:

(11) $$d\left(P_{c},L\right)=\frac{\left|-\left(z_{r}-z_{l}\right)x_{c}+\left(x_{r}-x_{l}\right)z_{c}+\left(z_{r}-z_{l}\right)x_{l}-\left(x_{r}-x_{l}\right)z_{l}\right|}{\sqrt{{\left(z_{r}-z_{l}\right)}^{2}+{\left(x_{r}-x_{l}\right)}^{2}}}$$

In case the ground projected coordinates *P_f_* of both feet overlapped, the above point-to-line distance can be simplified to a point-to-point Euclidean distance ‖*P_c_*−*p_f_*‖. With the availability of the sway distance *d*, the fall risk related to the body sway *p*(*sway*) was estimated as:

(12) $$p(\text{sway})=1-e^{-d^{2}(P_{c},L)/\alpha_{5}}$$

As a special case, when a toddler sat on a chair, the fall risk should not increase even if the ground-projected skeletal centroid was not located on the base-of-support line. Therefore, the body sway module was disabled if a sitting posture was detected by the SVM as described in Section 3.1.

In addition to the body sway, the body lean provides another important clue for the balancing. As shown in Figure 5b, the body lean was defined as the 3D angle between the body spine vector $\vec{\text{spine}}$ and the normal vector *n⃑* of the floor plane. The larger the angle between these two vectors was, the higher the fall risk should be. The spine vector was approximated by the 3D vector directing from the skeletal joint HEAP_CENTER to another skeletal joint SHOULDER_CENTER. Because the cosine of the angle *θ_h_* between the body spine vector and the floor normal vector was equal to the normalized inner product of these two vectors, the fall risk based on the body lean *p*(*lean*) was computed as:

(13) $$\begin{array}{l} \theta_{h}=\arccos(\frac{\vec{\text{spine}}\cdot \vec{n}}{\vec{\left\|\text{spine}\right\|}\cdot \left\|\vec{n}\right\|})\\ p(\text{lean})=1-e^{-\theta_{h}^{2}/\alpha_{6}} \end{array}$$

### 3.4. Altitude

The key to prevent falls from a substantial height is to beware of the body altitude above the ground. The body altitude was defined as the distance from the lowest skeletal joint (usually the FOOT_LEFT or FOOT_RIGHT) to the floor plane. The higher the body altitude was, the higher the fall risk should be. The 3D floor plane *F* was estimated based on the depth image and defined in the following equation:

(14) Ax+By+Cz+D=0

**Figure 6.**
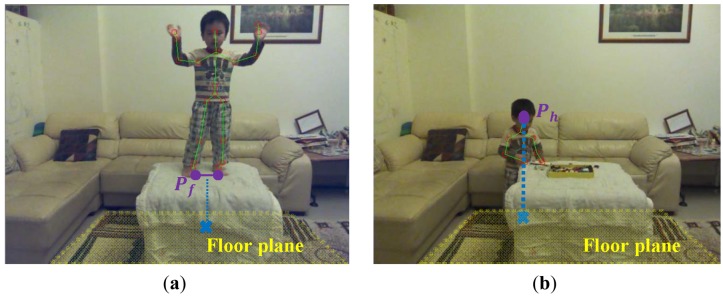
Altitude module (**a**) foot altitude and (**b**) head altitude.

The cosine of the angle *θ_k_* between the Kinect's vertical axis $\vec{\left(0,1,0\right)}$ and the floor normal vector $\vec{\left(A,B,C\right)}$ was equal to the normalized inner product of these two vectors. The cos(*θ_k_*) was used to perform the 3D scalar projection of the Kinect's vertical displacement vector to the floor normal vector. Supposing that the 3D midpoint of the locations of the skeletal joints of two feet (skeletal joints FOOT_LEFT and FOOT_RIGHT) was *P_f_* with coordinate (*x_f_*, *y_f_*, *z_f_*) as shown in Figure 6a, the distance from the point *P_f_* to the floor plane *F* was computed using the following equation:

(15) $$d(P_{f},F)=\left|Ax_{f}+By_{f}+Cz_{f}+D\right|\cdot \cos\theta_{k}=\left|Ax_{f}+By_{f}+Cz_{f}+D\right|\cdot \frac{\vec{\left(0,1,0\right)}\cdot \vec{\left(A,B,C\right)}}{\left\|\vec{\left(A,B,C\right)}\right\|}$$

Therefore, the fall risk related to the foot altitude *p*(*foot_altitude*) was estimated as:

(16) $$p(\text{foot}\_\text{altitude})=1-e^{-d^{2}(P_{f},F)/\alpha_{7}}$$

As a special case, when a toddler sat on a sofa, the fall risk should not increase even if the feet were above the floor. Therefore, the foot altitude module was disabled if a sitting posture was detected by the SVM as described in Section 3.1.

In case the feet were visual occluded as shown in Figure 6b, the human head altitude was an alternative for the fall risk assessment. A human body height *H* was approximated by summing the lengths of a set of bones (as shown as the connected blue line segments in Figure 2) in the detected skeleton. Supposing that the detected person stood in a upright pose and the location of the skeletal joint HEAD was *P_h_* with coordinate (*x_h_*, *y_h_*, *z_h_*), the distance from the lowest possible skeletal joint to the floor plane *F* was computed using the following equation:

(17) $$d(P_{h},F)=\left|Ax_{h}+By_{h}+Cz_{h}+D\right|\cdot \cos\theta_{k}-H$$

Similarly, the fall risk based on the head altitude *p*(*head*_*altitude*) was measured as:

(18) $$p(\text{head}\_\text{altitude})=\{\begin{array}{ll} 1-e^{-d^{2}(P_{h},F)/\alpha_{8}} & ,\text{if}\;d>0\\ 0 & ,\text{otherwise} \end{array}$$

## 4. Parameter Determination and Multi-Modal Fusion

**Figure 7.**
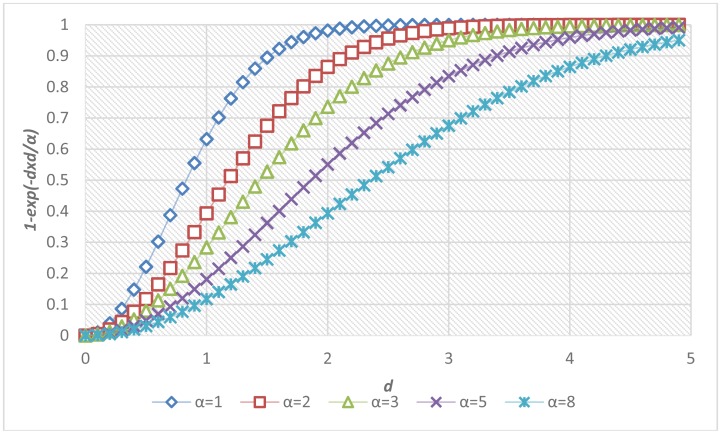
Relationship between the distance *d* and its fall risk *p*(*d*) with respect to different values of parameter α.

The determination of parameters was critical to the accuracy of the proposed fall risk assessment and alarm-triggering system. A local optimization was applied to determine the parameter α inside each individual module, and a global optimization was performed to determine the parameters in the multi-modal fusion. Figure 7 shows the sigmoid curves describing the relationships between the fall risk *p*(*d*) and the distance *d* regarding different values of the local parameter α. For each module, twenty video clips (3 s in each clip, 1,800 frames in total) containing normal ADL or corresponding fall-prone actions (such as run, jump, body sway, foot and head altitude) to various extents were captured. To acquire the ground truths of fall risks, each video was evaluated by a childcare expert using the questionnaire in Table 3. The local parameter α*_i_* in the *i*-th module was determined by fitting the twenty temporal means of the estimated fall risks $\left(1-e^{-d_{i}^{2}/\alpha }\right)$ to the ground truth fall risks (*p_i_*) according to the method of least squares. The fitting problem can be expressed as an over-determined system with a set of linear equations:

(19) $$\begin{array}{cc} \left[\begin{array}{c} -\ln(1-p_{i,1})\\ -\ln(1-p_{i,2})\\ :\\ -\ln(1-p_{i,20}) \end{array}\right]\cdot \alpha_{i} & =\left[\begin{array}{c} d_{i,1}^{2}\\ d_{i,2}^{2}\\ :\\ d_{i,20}^{2} \end{array}\right] \end{array}$$

Accordingly, the local parameter α*_i_* for the *i*-th module was chosen as the one that minimizes the sum of squared differences between the measured fall risks and their corresponding ground truths.

The overall fall risk was measured by integrating the measured fall risks from all modules. The goal of the multi-modal fusion was to increase precision and enhance reliability based on redundant information gathered from multiple modules [23]. If a module was disabled or failed to locate the required skeletal parts, its fall risk was set to zero. To train the global parameters in the multi-modal fusion, a Kinect captured 200 video clips (about 18,000 color and depth frames) of which half were used for training (training set) and the other half were used for testing (testing set). Each video lasted about three seconds and contained a toddler performing a normal ADL, a distinct fall-prone action, or a random combination of actions (such as simultaneously swaying and jumping, or climbing at an altitude) at home. To obtain the ground truths of fall risks *p_GT_*, each video clip was evaluated by a childcare expert using the questionnaire in Table 3. The input vector of the multi-modal fusion contained the temporal means of the estimated fall risks from *n* behavioral modules: {*p*_1_, *p*_2_,…, *p_n_*} (*n* = 8 in our case). The output of the multi-modal fusion was a Boolean decision of whether to trigger an alarm or not. The multi-modal fusion can be made using either one of the following two approaches.

### 4.1. Thresholding the Weighted Mean of the Estimated Fall Risks from All Modules

Supposing that the fall risks from *n* modules were sorted in a descending ordered list {*p*_(1)_, *p*_(2)_,…, *p*_(_*_n_*_)_} in that *p*_(1)_ was the maximal fall risk and *p*_(_*_n_*_)_ was the minimal fall risk, an intuitive multi-modal fusion was just to take the maximum or the mean of the fall risks among all modules. A more sophisticated choice was to take a weighted average in that the modules with higher fall risks received bigger weights. All these choices can be generalized in the following equation:

(20) $$\begin{array}{ccc} p_{\text{overall}}=\frac{1}{1-\beta^{n}}\left[p_{\left(1\right)}\cdot (1-\beta )+\overset{n}{\underset{i=2}{\text{∑}}}p_{\left(i\right)}\cdot (\beta^{i-1}-\beta^{i})\right] & , & 0\leq \beta <1 \end{array}$$

**Figure 8.**
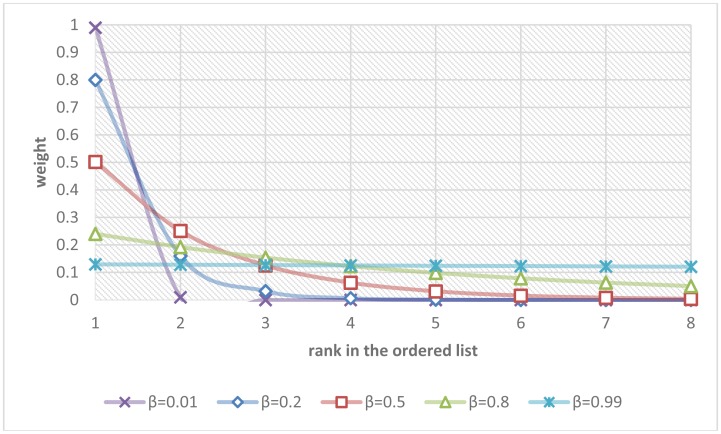
Relationship between the rank in the ordered list and its weight with respect to different values of parameter β.

The parameter β controls the rate of diminishing weights. Figure 8 shows the curves describing the relationships between the ranks in the ordered list and their weights regarding different values of the parameter β.

If the parameter β was set to zero, the overall fall risk was equal to the maximal fall risk *p*_(1)_. If the parameter β was set to 0.5, the overall fall risk was close to *p*_(1)_/2 + *p*_(2)_/4 + *p*_(3)_/8 + … + *p*_(_*_n_*_)_/2*^n^*. If the parameter β approached one, the overall fall risk approximated the mean of the fall risks from all modules, *i.e.*, (*p*_(1)_ + *p*_(2)_ + *p*_(3)_ + … + *p*_(_*_n_*_)_)/*n*. The parameter β were determined by fitting the estimated overall fall risks *p_overall_* to the ground truths of fall risks *p_GT_* using a recursive one-dimensional grid-based search to minimize the fitting errors over the training set of video clips. Finally, a simple thresholding was employed to make the final Boolean decision of the alarm triggering. The parameter *T* controlled the sensibility of the system and can be adaptively adjusted by users (*T* = 0.5 in our experiments):

(21) $$\text{Alarm}\_\text{triggering}=\{\begin{array}{ll} 1 & ,\text{if}\;p_{\text{overall}}>T\\ 0 & ,\text{otherwise} \end{array}$$

### 4.2. Classification by SVM Based On the Estimated Fall Risks from All Modules

The multi-modal fusion of fall risks for the alarm triggering can also be formalized as a multi-dimensional classification by using a SVM. For the *j*-th video clip in the training set with *l* video clips (*l* = 100 in this case), a *n*-dimensional vector *x_j_* contained the estimated fall risks from *n* modules: {*p*_1_, *p*_2_,…, *p_n_*} (*n* = 8 in our case) and a Boolean flag *y_j_* indicated the decision of the alarm triggering based on the ground truth fall risk *p_GT_*:

(22) $$y_{j}=\{\begin{array}{cc} +1 & ,\text{if}\;p_{GT}>T\\ -1 & ,\text{otherwise} \end{array}$$

The parameter *T* controls the sensibility of the system and can be adaptively adjusted by users (*T* = 0.5 in our experiments). Given a set of labeled pairs (*x_j_*, *y_j_*), the SVM became an optimization problem to find an optimal separating hyperplane with maximum margins. The training vectors *x_i_* were transformed into a higher dimensional space by the kernel function *K*. After experimenting a linear kernel and various nonlinear kernels, we found that the radial basis function (RBF) provided the most accurate classification of the alarm triggering:

(23) $$K(x_{j},x_{k})=\exp(-\gamma {\left\|x_{j}-x_{k}\right\|}^{2})$$

The penalty parameter *C* and RBF parameter γ were determined by a recursive two-dimensional grid-based search to maximize the cross-validation accuracy over the training set of video clips. The final Boolean decision of the alarm triggering was made by:

(24) $$\text{Alarm}\_\text{triggering}=\{\begin{array}{ll} 1 & ,\text{if}(w^{T}\phi (x_{j})+b)\;\text{is positive}\\ 0 & ,\text{otherwise} \end{array}$$

## 5. Results and Discussion

The computational platform of the proposed fall risk assessment and early-warning system was a PC equipped with a 3.4 GHz Intel Core i7 CPU and the 64-bit Windows 7 operating system. The program was coded in C language utilizing the Kinect SDK, LIBSVM [24], and OpenCV [25] library. The performance of the proposed fall risk assessment system was evaluated based on the aforementioned testing set of 100 video clips captured by a Kinect at home, composed of more than 9000 color and depth frames. Among those, 50 video clips contained various arranged scenarios with different fall-prone actions, and 50 video clips contained normal ADL in home environments.

This paper focused on the identification of behavioral factors instead of environmental factors. However, effort was made to protect the toddler during our experiments. A thick carpet or blanket was laid on the floor when necessary. Sharp edges on furniture were covered by silicon corner protectors. A soft pad was installed on top of the table. Cushions or pillows were placed over all spots at risk for potential collisions. Moreover, two adults stood by either side of the toddler for immediate response to any possible slips. Because our goal was to develop an early-warning system for fall risk assessment, no real falls happened during our experiments.

**Figure 9.**
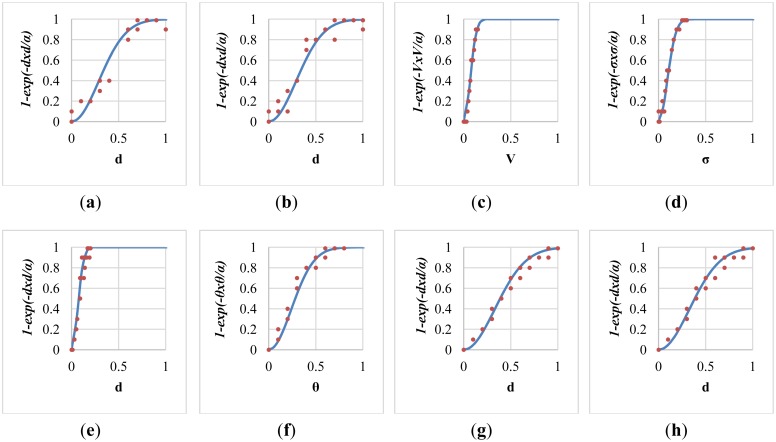
Least square error optimization to determine the local parameter α in each module. (**a**) push-up climb; (**b**) pull-up climb; (**c**) rush-running; (**d**) high-jumping; (**e**) body sway; (**f**) body lean; (**g**) foot altitude; and (**h**) head altitude.

After the local parameter optimization based on the method of least squares, the fitted sigmoid curve in each individual fall-prone behavioral module was shown in Figure 9. Twenty training data for the curve fitting were marked as red dots in each sub-figure. Specifically, the determined value of the local parameters α for these modules were 0.167, 0.168, 0.009, 0.017, 0.008, 0.115, 0.217, and 0.214 respectively, with average errors of 0.065, 0.058, 0.036, 0.039, 0.048, 0.044, 0.037, and 0.044 respectively. The modelling error of the module of push-up climb was relatively high (ε = 0.065) because it tended to be confused with other normal ADL.

**Figure 10.**
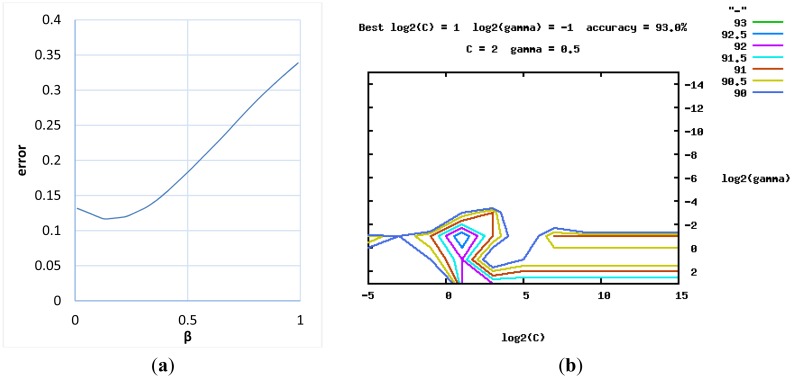
Global parameter determination based on a recursive grid-based search. (**a**) diminishing parameter β; and (**b**) SVM's parameters: *C* and gamma γ.

The global parameters in the multi-modal fusion were optimized based on the aforementioned training set of 100 video clips. For the first multi-modal fusion scheme of the weighted mean thresholding, Figure 10a plots the 1D grid-based search that determined the best value of the diminishing parameter (β = 0.14) with the minimal modelling error (ε = 0.117). Besides, it was noted that taking the maximum (β = 0, ε = 0.133) was much better than taking the mean (β = 0.99, ε = 0.339) in the multi-modal fusion. For the second multi-modal fusion scheme of the SVM classification, Figure 10b shows the 2D grid-based search that discovered the values of the SVM's parameters (*C* = 2 and γ = 0.5) with the maximal cross-validation accuracy. The grid-based search was an iterative algorithm that tried to find out better parameters with higher cross-validation accuracy in each iteration. Each color contour in the figure represented the range of parameters with the same cross-validation accuracy in a single iteration.

All fall-prone video clips manually marked by a childcare expert (ground truth fall risk *p_GT_* > 0.5 in our experiments) were called positive samples (*P*), and all other video clips were called negative samples (*N*). The true positive (*TP*) was defined as the number of triggered alarms in positive samples, the false positive (*FP* or false alarm) was defined as the number of triggered alarms in negative samples, the false negative (*FN* or miss-detections) was defined as the number of positive samples that did not trigger alarms, and the true negative (*TN*) was defined as the number of negative samples that did not trigger alarms. Accordingly, the *accuracy rate* was measured as (*TP* + *TN*)/(*P* + *N*). Table 5 showed the confusion matrices of the proposed early-warning system with the multi-modal fusion using either a weighted mean thresholding or a SVM classification. In summary, the former scheme (weighted mean thresholding) achieved an accuracy rate of 92%, and the latter scheme (SVM classification) achieved an accuracy rate of 91%. A closer look at the mistakes of the alarm triggering revealed that most false alarms resulted from the localization errors of the skeletal parts, and most miss-detections were caused by the failures of the skeletal tracking.

**Figure 11.**
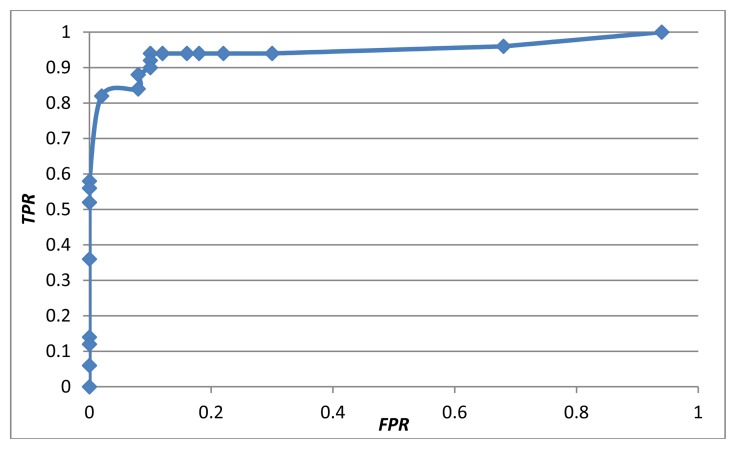
ROC curve of the proposed early-warning system.

A true positive rate (*TPR*) was defined as the number of true positives (*TP*) divided by the number of positive samples (*P*). A false positive rate (*FPR*) was defined as the number of false positives (*FP*) divided by the number of negative samples (*N*). Figure 11 shows the receiver operating characteristic (ROC) curve of the proposed early-warning system using the multi-modal fusion of the weighted mean thresholding. To balance the trade-off between the *TPR* and *FPR* based on the ROC curve, the threshold *T* was set to 0.5, achieving a *TPR* of 94% and a *FPR* of 10%.

As far as we know, the proposed system is the first early-warning and fall risk assessment system for toddlers using a Kinect at home. The closest method with a similar goal was Na's work [14] which tracked a toddler using region splitting and merging based on color images captured by a webcam. Subsequently, they examined whether the lowest part of the human contour was on, near or leaving the floor area boundary to identify fall risks. For comparison purposes, we implemented their algorithms and performed evaluations using our testing set of video clips. To focus on the behavioral factors, the ground clutter detection was not implemented in both systems. Compared with their system, which had an accuracy rate of 65%, the proposed system has an accuracy rate of 92%, making it much more reliable. Table 5 compared the confusion matrices of the proposed early-warning system with Na's work. To compare the robustness of the proposed system with Na's work in more challenging conditions, 50 video clips were captured with self-occlusions and partial occlusions. Among those, 25 video clips contained various arranged scenarios with different fall-prone actions, and 25 video clips contained normal ADL in home environments. The accuracy rate was 84% using the proposed system, compared to 62% using Na's algorithm. The improvement in terms of accuracy rate resulted mainly from two factors. First, their method computed 2D optical flows solely based on color images, whereas our method considered 3D skeletal features with the availability of the Kinect's depth images. Second, they considered only the relationship between the toddler's lowest body part and the floor area boundary, whereas we utilized eight fall-prone toddler's behavior modules based on four criteria: posture, motion, balance, and altitude.

For simplicity, our experiments considered a single toddler in the Kinect's field of view and tracked only one skeleton at a time. With minor modifications, the proposed system can track up to two toddlers simultaneously and assess the fall risks individually. However, a profile of the execution time showed that the body skeleton tracking took up a major portion of the CPU time and was the bottleneck of the proposed system. Generally, the proposed system run at 20 frames per second (FPS) for a single skeleton, and 13 FPS for two skeletons.

The field of view (FOV) of a Kinect is 43° vertically and 57° horizontally. Kinect is also equipped with a built-in motorized platform that can be programmed with a vertical tilt range of ±27° to extend the vertical FOV dynamically. To further extend the viewing perspectives, the Kinect SDK supports up to four Kinects simultaneously connected to a single computer. Nevertheless, the infrared light patterns emitted by multiple Kinects can interfere with each other and reduce the accuracy of skeletal tracking. Moreover, the computation loads of the tracking of multiple skeletons can also slow down the execution speed. The Kinect's specifications show that the optimal distance between a Kinect and its target is about 0.8–4.0 m, and the possible working distance is about 0.4–8.0 m. Our experiences during the experiments indicated that the Kinect skeleton tracking was reliable in a 3D frustum with a size of about 4 m (*x*-axis) × 3 m (*y*-axis) × 3 m (*z*-axis). By choosing a proper installation position and angle, a single Kinect should be able to cover the playing area in a small living room or a typical kid's room for most families.

**Figure 12.**
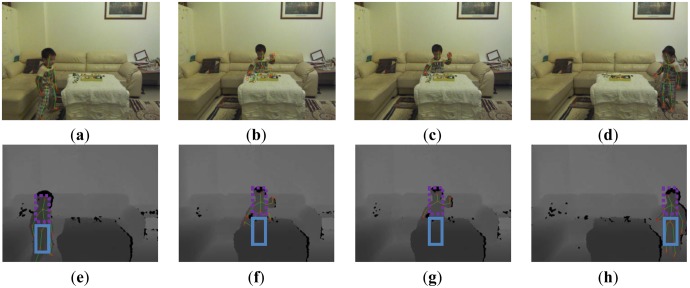
Partial occlusion on the human lower body. (**a**) before occlusion; (**b**) entering occlusion: default mode; (**c**) under occlusion: seated mode; (**d**) leaving occlusion. And (**e**)–(**h**) respective depth images. The average depth values in the dashed rectangle (for the upper body) and in the solid rectangle (for the lower body) were compared.

There are two kinds of occlusions. First, the self-occlusion (some body parts were hidden from the viewpoint of the camera) was handled by the Kinect SDK of the skeleton tracking. Because the depth information was missing at the hidden parts, the Kinect SDK used a learner discriminative approach to handle the self-occlusions [19]. Temporal and kinematic coherence can also be incorporated to infer the locations of the missing parts. Obdrzalek *et al.* [26] have reported the accuracy and robustness of the Kinect's skeleton tracking with respect to self-occlusions. Second, partial occlusions happened when some body parts were occluded by closer objects, such as furniture. We concentrated on handling the partial occlusions that usually happened on the human lower body, as shown in Figure 12. The Kinect skeleton tracking provided a special tracking mode (called seated mode) that focused on the tracking of the human upper body. Compared to the default mode that tracked twenty joints, the seated mode tracked only ten joints on the upper body and was optimized to track people whose lower body was not entirely visible, or who were seated on a chair or couch. Our system switched between these two tracking modes automatically by analyzing the skeleton area in the depth images to detect occlusions. The depth values in the skeleton area were summed together along the *x* axis to generate a horizontal projection profile. If the average depth of the lower body was reduced suddenly (as shown in Figure 12b), it indicated that the lower body was occluded and the skeleton tracking system switched to the seated mode to concentrate on tracking the upper body (as shown in Figure 12c). Conversely, if the average depth of the lower body returned to the original range that was similar to the depth of the upper body, it indicated a leaving of occlusion state and the skeleton tracking system switched to the default mode to track the complete body (as shown in Figure 12d). Switching between these two modes prevented drift-over-time errors of the skeleton tracking in scenarios with lower body occlusions. However, the body sway and foot altitude modules were disabled in the seated mode (as shown in Figure 1) and the accuracy rate of the alarm triggering degraded in situations with lasting occlusions (from 92% to 84% in our experiments).

## 6. Conclusions

We proposed an early-warning fall assessment system for toddler healthcare at home. Depth images captured by a Kinect system were analyzed to locate 3D human skeletal joints and estimate a floor normal vector. Subsequently, eight fall-prone behavioral modules were designed for fall risk assessments based on four essential criteria: posture, motion, balance, and altitude. Fall risks measured by all modules were integrated using a multi-modal fusion based on either a weighted mean thresholding or a SVM classification. Both local and global parameters were trained by a set of video clips, and the proposed fall assessment system was tested by another set of video clips. Experimental results demonstrated that the fusion by the weighted mean achieved an accuracy rate of 92%, and fusion by SVM achieved an accuracy rate of 91%. The frame rate of the proposed early-warning childcare system was around 20 FPS.

The limitation of the proposed work is three-fold. First, current Kinect's skeleton tracking failed to handle some irregular body poses such as lying or upside-down. Second, despite the supports of up to four Kinects simultaneously connected to a single computer, the issues of infrared interference, calibration, synchronization, computation load, and gallery guarding remain to be solved in the future. Third, even though it was possible to track up to two toddlers at a time, their fall risks were assessed independently without considering the interaction between them. In the future, we propose to explore the possibilities of developing more fall-prone behavioral modules by taking toddlers' interactions into account.

## Figures and Tables

**Table 1.** Comparison of various wearable sensors for fall and pre-impact detection.

**Methods**	[2]	[8]	[3]	[9]	[10]	[11]
**Hardware**	Accelerometer		Gyroscope	Accelerometer + Gyroscope		
		
**Detection stage**	Fall	Pre-impact	Fall	Pre-impact	Pre-impact	Pre-impact
**Targets**	Elder	Elder	Elder	Elder	Elder	Elder
**Algorithm**	Vertical angle	HMM + SVM	Angular velocity + Acceleration + trunk angle	SVM	Vertical velocity	Thigh + Torso angular data
**Intrusion**	Yes	Yes	Yes	Yes	Yes	Yes
**Tracking**	No	No	No	No	No	No
**Shadow handling**	Yes	Yes	Yes	Yes	Yes	Yes

**Table 2.** Comparison of fall detection approaches using distinct kinds of cameras.

**Methods**	[4]	[5]	[14]	[15]	[6]	[16]	[7]	[17]	This work
**Hardware**	Infrared Camera		Color Camera			Depth Camera		Kinect	
			
**Detection Stage**	Fall	Fall	Track off floor	Behavior modeling	Fall	Fall	Fall	Get-up event	Early-warning
**Targets**	Elder	Elder	Toddler	Infant	Elder	Elder	Elder	Patient	Toddler
**Algorithm**	Neural network	SVM	Optical flow	Bayesian network	MHI + Shape change	MBR ratio + Velocity + Depth variation	Skeleton + Floor	MHI + HOG + HOF	Skeleton + SVM + Floor
**Intrusion**	No	No	No	No	No	No	No	No	No
**Tracking**	Yes	Yes	Yes	Yes	Yes	Yes	Yes	Yes	Yes
**Shadow handling**	Yes	Yes	No	No	No	Yes	Yes	Yes	Yes

**Table 3.** Questionnaire completed by a childcare expert to determine the ground truths of fall risks in each video.

very safe	← …← …← …←				not sure	→ …→ … → … →				very fall-risky
*p* = 0	0.1	0.2	0.3	0.4	0.5	0.6	0.7	0.8	0.9	*p* = 1

**Table 4.** Various behaviors in daily life of a toddler at home. The last row indicates the criteria that are useful to classify each behavior as fall-risky or safe.

**Classification**	**Safe**					**Fall-risky**				
		
**Behaviors of toddlers in daily life**	sitting on floor or chair	standing on floor	walking, strolling	jumping rope	dancing, hula hooping	rush running	high jumping	tumbling, crawling	climbing table or stair	standing on chair or table
	
**1. Posture**	*sit*	*upright*	*upright*	*upright*	*upright*	*upright*	*upright*	*varying*	*climb*	*upright*
**2. Motion**	*low*	*low*	*medium*	*medium*	*medium*	*high*	*high*	*medium*	*medium*	*low*
**3. Balance**	*lean*	*good*	*good*	*good*	*lean*	*lean*	*good*	*lean + sway*	*lean + sway*	*good*
**4. Altitude**	*low*	*low*	*low*	*medium*	*low*	*low*	*high*	*low*	*high*	*high*
	
**Criteria**	124	1234	134	13	14	23	24	13	134	4

**Table 5.** Confusion matrices of the alarm triggering based on the fall risk assessments of toddlers. (**a**) first proposed scheme: weighted mean thresholding; (**b**) second proposed scheme: SVM classification; and (**c**) Na's method [14].

**GT Decision**	**Positive**	**Negative**	**GT Decision**	**Positive**	**Negative**	**GT Decision**	**Positive**	**Negative**
		
positive	46	5	positive	47	5	positive	16	1
negative	4	45	negative	3	45	negative	34	49
		
	(**a**)			(**b**)			(**c**)	
